# Implementing Multifactorial Risk Assessment with Polygenic Risk Scores for Personalized Breast Cancer Screening in the Population Setting: Challenges and Opportunities

**DOI:** 10.3390/cancers16112116

**Published:** 2024-05-31

**Authors:** Meghan J. Walker, Kristina M. Blackmore, Amy Chang, Laurence Lambert-Côté, Annie Turgeon, Antonis C. Antoniou, Kathleen A. Bell, Mireille J. M. Broeders, Jennifer D. Brooks, Tim Carver, Jocelyne Chiquette, Philippe Després, Douglas F. Easton, Andrea Eisen, Laurence Eloy, D. Gareth Evans, Samantha Fienberg, Yann Joly, Raymond H. Kim, Shana J. Kim, Bartha M. Knoppers, Aisha K. Lofters, Hermann Nabi, Jean-Sébastien Paquette, Nora Pashayan, Amanda J. Sheppard, Tracy L. Stockley, Michel Dorval, Jacques Simard, Anna M. Chiarelli

**Affiliations:** 1 Ontario Health (Cancer Care Ontario), Toronto, ON M5G 2L3, Canada; 2 Dalla Lana School of Public Health, University of Toronto, Toronto, ON M5S 1A1, Canada; 3 CHU de Québec-Université Laval Research Center, Queébec City, QC G1V 4G2, Canada; 4 Centre for Cancer Genetic Epidemiology, Department of Public Health and Primary Care, School of Clinical Medicine, University of Cambridge, Cambridge CB1 8RN, UK; 5 Department for Health Evidence, Radboud University Medical Center, 6525EP Nijmegen, The Netherlands; 6 Department of Family Medicine and Emergency Medicine, Faculty of Medicine, Université Laval, Québec City, QC G1V 0A6, Canada; jean-sebastien.paquette@fmed.ulaval.ca; 7 Department of Physics, Engineering Physics and Optics, Faculty of Science and Engineering, Université Laval, Quebec City, QC G1V 0A6, Canada; 8 Sunnybrook Health Science Center, Toronto, ON M4N 3M5, Canada; 9 Québec Cancer Program, Ministère de la Santé et des Services Sociaux, Quebec City, QC G1S 2M1, Canada; 10 Division of Evolution Infection and Genomic Sciences, The University of Manchester, Manchester M13 9PL, UK; 11 Centre of Genomics and Policy, McGill University, Montreal, QC H3A 0G1, Canada; 12 Princess Margaret Cancer Centre, Toronto, ON M5G 2M9, Canada; 13 Women’s College Research Institute, Toronto, ON M5G 1N8, Canada; 14 Department of Social and Preventive Medicine, Faculty of Medicine, Université Laval, Quebec City, QC G1V 0A6, Canada; 15 Université Laval Cancer Research Center, Quebec City, QC G1R 3S3, Canada; 16 Department of Applied Health Research, Institute of Epidemiology and Healthcare, University College London, London WC1E 6BT, UK; 17 Division of Clinical Laboratory Genetics, University Health Network, Toronto, ON M5G 2C4, Canada; 18 Department of Laboratory Medicine and Pathobiology, University of Toronto, Toronto, ON M5S 1A8, Canada; 19 Faculty of Pharmacy, Université Laval, Quebec City, QC G1V 0A6, Canada; 20 Department of Molecular Medicine, Faculty of Medicine, Université Laval, Quebec City, QC G1V 4G2, Canada

**Keywords:** breast cancer, breast cancer screening, risk assessment, polygenic risk score, risk stratification, implementation

## Abstract

**Simple Summary:**

The current approach to breast cancer screening, which is based on a person’s age, overlooks individual-level differences in breast cancer risk. As a result, many people are over- or under-screened according to their actual risk of breast cancer. Risk-stratified breast screening may overcome the limitations of age-based screening, but there are still many knowledge gaps regarding how best to implement it in the population setting. This study will generate the first Canadian evidence on the adoption of breast cancer risk assessment in the population setting, to support the future implementation of risk-stratified breast cancer screening. This study demonstrated that, while risk assessment for risk-stratified screening at the population level is feasible, an equity lens must be considered in implementation to ensure cancer-screening disparities are not widened.

**Abstract:**

Risk-stratified breast screening has been proposed as a strategy to overcome the limitations of age-based screening. A prospective cohort study was undertaken within the PERSPECTIVE I&I project, which will generate the first Canadian evidence on multifactorial breast cancer risk assessment in the population setting to inform the implementation of risk-stratified screening. Recruited females aged 40–69 unaffected by breast cancer, with a previous mammogram, underwent multifactorial breast cancer risk assessment. The adoption of multifactorial risk assessment, the effectiveness of methods for collecting risk factor information and the costs of risk assessment were examined. Associations between participant characteristics and study sites, as well as data collection methods, were assessed using logistic regression; all *p*-values are two-sided. Of the 4246 participants recruited, 88.4% completed a risk assessment, with 79.8%, 15.7% and 4.4% estimated at average, higher than average and high risk, respectively. The total per-participant cost for risk assessment was CAD 315. Participants who chose to provide risk factor information on paper/telephone (27.2%) vs. online were more likely to be older (*p* = 0.021), not born in Canada (*p* = 0.043), visible minorities (*p* = 0.01) and have a lower attained education (*p* < 0.0001) and perceived fair/poor health (*p* < 0.001). The 34.4% of participants requiring risk factor verification for missing/unusual values were more likely to be visible minorities (*p* = 0.009) and have a lower attained education (*p* ≤ 0.006). This study demonstrates the feasibility of risk assessment for risk-stratified screening at the population level. Implementation should incorporate an equity lens to ensure cancer-screening disparities are not widened.

## 1. Introduction

Breast cancer is a leading cause of morbidity and mortality in Canada [1]. The evidence demonstrates that breast screening with mammography is effective for reducing breast cancer mortality [2,3]. Breast cancer screening guidelines use an age-based approach, and until recently, many recommended mammography every 2–3 years for people aged 50–74 [2,4]. 

The age-based approach to screening overlooks population heterogeneity in breast cancer risk. As a result, many people are over- or under-screened according to their actual risk. Emerging evidence suggests that breast screening may need to begin earlier for some racial groups [5,6]. Breast screening guidelines in the United States were recently updated to recommend biennial mammography for people aged 40–74 [7], and guidelines in Canada are being updated to reflect modern evidence. 

Given the substantial limitations of age-based breast screening, there is considerable international attention on moving to an approach that incorporates a more comprehensive understanding of breast cancer risk [8,9]. Under this more comprehensive risk-based approach, breast screening recommendations (e.g., start age, screening modality, interval) are tailored to an individual’s risk for breast cancer estimated via a multifactorial risk assessment. Multifactorial risk assessment refers to the estimation of the risk of developing breast cancer within a specified time, considering the combined effects of genetic and non-genetic risk factors, such as rare high- and moderate-penetrance genetic variants (e.g., *BRCA1, BRCA2, PALB2, ATM*), common low-penetrance genetic variants, a family history of breast and other cancers, breast density and reproductive, hormonal, anthropometric and lifestyle factors [8]. Modeling studies suggest that risk-based breast screening is more cost-effective and can optimize the benefit–risk ratio by improving cancer detection, while reducing potential harms [10,11]. Numerous tools have been developed for risk estimation, including several which have more recently incorporated polygenic risk scores (PRS) to account for the multiplicative effects of common genetic variants associated with breast cancer risk [12,13]. A review of risk assessment tools demonstrated that discriminative accuracy may be improved by combining the PRS with genetic and non-genetic risk factors [14].

There is still much to be learned about the optimal methods of delivering risk-based breast screening on a large scale before it can be fully implemented in the population. To address this, an international network of studies is evaluating efficacy, effectiveness, cost-effectiveness, feasibility, acceptability and health system readiness, as well as social, ethical and legal issues related to risk prediction and communication [15,16,17,18,19,20,21]. 

The PERSPECTIVE I&I project is generating the first real-world evidence on the delivery of risk-stratified screening based on multifactorial risk assessment within organized breast screening programs in Canada. The objectives of this prospective cohort study that was undertaken within the PERSPECTIVE I&I project were to (i) describe those who participated in multifactorial risk assessment; and (ii) evaluate the effectiveness and cost of various methods of collecting breast cancer risk factor information to facilitate risk estimation.

## 2. Materials and Methods

### 2.1. Study Design and Setting

The PERSPECTIVE I&I project has been previously described [21]. Briefly, the goal is to improve breast cancer risk assessment and identify optimal approaches for implementing risk-based screening and prevention in Canadian health systems through four interconnected activities: (i) the identification and validation of novel moderate- to high-risk breast cancer susceptibility genes through a whole exome sequencing case–control study to support the development of a comprehensive multi-gene panel test; (ii) the improvement, validation and adaptation of a comprehensive risk prediction web tool for the Canadian context; (iii) the development and piloting of a socio-ethical framework to support the implementation of risk-based breast screening at the population level; and (iv) economic simulation modeling to optimize the implementation of risk-stratified breast cancer screening.

As part of the third activity, a large prospective cohort study was conducted, which recruited eligible people from two study sites in Canada to undergo a multifactorial breast cancer risk assessment, including the PRS, and receive risk-stratified screening and prevention recommendations. 

### 2.2. Study Population and Recruitment 

Females aged 40–69 in Ontario and Quebec who had a previous mammogram were invited to participate in the prospective cohort study from July 2019 to December 2021. Ontario and Quebec are Canada’s two most populous provinces, both with publicly funded healthcare systems and well-established organized breast cancer screening programs. The two study sites had distinct clinical and operational policies and procedures, which were reflected in the study eligibility criteria and recruitment strategies. Those with a personal history of breast, ovarian, or pancreatic cancer or mastectomy, a known high risk of breast cancer, or who previously had genetic testing or counselling for breast cancer were not eligible. Participants were asked to complete three questionnaires, provide a saliva sample and consent to the collection of their most recent mammogram report to facilitate the multifactorial risk assessment. 

In Ontario, letters of invitation were mailed to 10,145 people aged 50–69 who had a mammogram at one of six Ontario Breast Screening Program (OBSP) sites that offer average- and high-risk screening services in the Waterloo Wellington, Hamilton Niagara Haldimand Brandt, Toronto Central and South East regions of the province. Participants aged 40–69 were also recruited via advertisements through mammography centres, primary care providers, webpages, newsletters and social media. 

In Quebec, recruitment strategies included advertisement via mammography centers, traditional (e.g., television) and social media, email listservs of affiliate organizations and a study website. Participants were required to register for the study online, have a regular primary care provider and have had a previous mammogram in one of 13 screening centers in the Lanaudière or Capitale-Nationale regions of the province.

### 2.3. Data Collection

#### 2.3.1. Questionnaires

The entry questionnaire captured risk factor information required by the validated Breast and Ovarian Analysis of Disease Incidence and Carrier Estimation Algorithm (BOADICEA) model [12,13,22,23] used in the CanRisk prediction tool [24], including a first- or second-degree family history of breast, ovarian, prostate and pancreatic cancer, and hormonal, anthropometric and lifestyle factors. In Ontario, questionnaires could be completed online, on paper or by telephone with study personnel. In Quebec, participants completed questionnaires online. The first questionnaire was completed at study entry from July 2019 to June 2022. Study personnel contacted a subset of participants to verify or correct missing or unusual response values for breast cancer risk factors in the entry questionnaire to maximize the accuracy of the risk estimation. The second questionnaire was completed at the time of the risk communication and the third was sent approximately one year following the risk communication.

The following breast cancer risk factors were self-reported by participants in the entry questionnaire and categorized for risk estimation and analysis according to the definitions used in the BOADICEA risk model included in the CanRisk tool: age at study entry, height, weight, age at menarche (<11, 11, 12, 13, 14, 15 or >15 years), menopausal status (premenopausal, postmenopausal [periods stopped for ≥6 months]), age at menopause (<40; 40–44; 45–49; 50–54; or ≥55), oral contraceptive use (never, former or current user), menopausal hormone therapy use (never, former or current user), parity (nulliparous, 1 birth, 2 births, >2 births), age at first live birth (<20; 20–24; 25–29; or ≥30), alcohol intake (0, >0 to <5, 5 to <15, 15 to <25, 25 to <35, 35 to <45, or ≥45 g per day, based on Canadian standard drink volumes. Additional information on the calculation of alcohol intake is included in Appendix A.)

A medical history and breast cancer-screening practices, knowledge, attitudes, beliefs about breast cancer, screening and genetic counselling/testing, as well as psychosocial, general health and sociodemographic factors, were also collected. Sociodemographic and general health factors included the following: country of birth, ethnic/cultural origin, Indigenous identity, marital status, highest level of attained education, current employment status and overall health. The format of these questions in the entry questionnaire were based on questions contained in the Canadian Census and Canadian Community Health Survey. Responses from the questions on ethnic/cultural origin and Indigenous identity were used to create an additional derived variable on visible minority group membership according to the Statistics Canada definition [25].

#### 2.3.2. Saliva Samples and Polygenic Risk Scores

Participants were asked to provide a saliva sample using a collection kit, to provide a source of DNA for a clinical-grade Breast Cancer Genetic Risk Single Nucleotide Polymorphisms (SNPs) test. This test uses Next Generation Sequencing (NGS) methods to genotype 295 SNPs based on the 313-SNP breast cancer PRS identified in a prior PERSPECTIVE study [22,23]. The extraction of DNA from saliva samples and genetic testing were conducted in an accredited molecular lab at Princess Margaret Cancer Center. The PRS was calculated using an algorithm which summarizes the combined effects of the 295 SNPs [22]. The BOADICEA-specific parameters for this PRS have been published elsewhere [23]. Where the initial sample was insufficient, yielded inconclusive results or met the threshold for failed SNPs (≥3 random failed SNPs) and a PRS could not be calculated, participants were contacted to request a second sample.

#### 2.3.3. Breast Density

Breast Imaging Reporting and Data System (BI-RADS^®^) [26] categories of breast density are used in the CanRisk prediction tool for risk estimation. The most recent mammogram report for each participant was obtained and the mammographic breast density was abstracted by trained study personnel. Where available, the BI-RADS^®^ breast density was abstracted and recorded as A (almost entirely fatty), B (scattered density), C (heterogeneously dense) or D (extremely dense). 

In Ontario, mammogram reports were obtained by accessing the electronic hospital records of the 6 participating Ontario Breast Screening Program sites. The BI-RADS^®^ density was abstracted from mammogram reports onto a standardized form and entered into a study database. At the time of study recruitment, the Ontario Breast Screening Program recommended that participants with a percent mammographic density ≥75% be screened every year. For participants where the mammogram report specified an annual screening recommendation due to a high breast density, the breast density was abstracted as BI-RADS^®^ category D. For all other participants, the breast density included on the mammogram report was abstracted. On 26.7% of the mammogram reports, a BI-RADS^®^ category was not reported, and the breast density was reported as <75%. For the purposes of the risk assessment of these participants, the breast density was treated as missing and the age-specific mean BI-RADS^®^ breast density category was imputed for the risk estimation. This may have resulted in an under- or over-estimation of breast density for some participants. For the purposes of this analysis, a breast density reported as <75% was categorized as BI-RADS^®^ A/B/C, and a breast density reported as ≥75% was categorized as BI-RADS^®^ D. 

In Quebec, mammogram reports were obtained from participating screening centers, and breast density information was abstracted. Where the breast density was not reported or a mammogram report was not accessible, radiologists were contacted to review mammograms and provide the BI-RADS^®^ breast density, to minimize missing values. For participants where the breast density remained missing (e.g., the report was no longer available to review), the age-specific mean BI-RADS^®^ breast density category was imputed for risk estimation. This may have resulted in an under- or over-estimation of breast density for some participants. Of the 1642 mammogram reports requested, 12 (0.7%) were unavailable and 39 (2.4%) were >10 years old and thus not used for risk calculation. The BI-RADS^®^ density was available in 1466 (89.3%) reports, while it was not reported according to the BI-RADS^®^ classification or was missing in 125 (7.6%). Collaboration with radiologists resulted in obtaining the BI-RADS^®^ density in 123 (98.4%) of these cases, permitting 1589 (96.8%) to be used for risk calculation.

### 2.4. Breast Cancer Risk Estimation

To estimate participants’ risk, risk factor information, the standardized PRS (beta) and breast density were entered into the CanRisk web tool [21,22,23]. The CanRisk tool was released in November 2019 and is a web-based software to predict the risk of breast cancer, contralateral breast cancer, ovarian cancer and mutation carrier probabilities. It is based on the BOADICEA model and identifies at-risk individuals by combining various risk factors (genetic, lifestyle, hormonal, reproductive, a first- or second-degree family history of breast, ovarian, prostate and pancreatic cancer, and mammographic density) using risk prediction models to provide a personalized cancer risk assessment and risk stratification to optimize clinical management. While CanRisk considers the effects of rare pathogenic variants in moderate and high risk susceptibility genes, the presence of variants in these genes was treated as unknown for risk estimation in our study. This is because variants in these susceptibility genes were not tested for in the context of our study and individuals with a prior history of genetic counselling or testing were excluded. 

CanRisk was used to estimate an age-specific 10-year breast cancer risk, which was stratified into three risk categories based on risk groupings used in the OBSP and some other Canadian screening programs. The risk categories correspond to the remaining lifetime (RLT) risk for people aged 30–80 (anchored at age 30) and were defined as average (<15% RLT), higher than average (15% to <25% RLT) and high (≥25% RLT) (9). Risk was estimated without the PRS for participants with an inconclusive PRS. CanRisk version 1.0.4 was used for risk assessments completed on or before 31 January 2022, following which CanRisk version 2.0.0 was used.

### 2.5. Statistical Analysis 

A comparison of risk factors between study sites was examined using logistic regression adjusted for age at entry, while comparisons of sociodemographic and health-related characteristics were assessed using univariate logistic regression. Characteristics associated with the mode of questionnaire completion and risk factor verification status were assessed, using stepwise multivariate logistic regression. Generalizability was assessed by comparing the cohort to two representative Canadian samples with standardized differences (Appendix A). The per-participant costs of a multifactorial risk assessment were estimated, considering all materials and infrastructure, mailing and labor. Analyses were performed using SAS version 9.4 [27], using two-tailed tests with a 5% threshold for statistical significance.

## 3. Results

**Figure 1:**
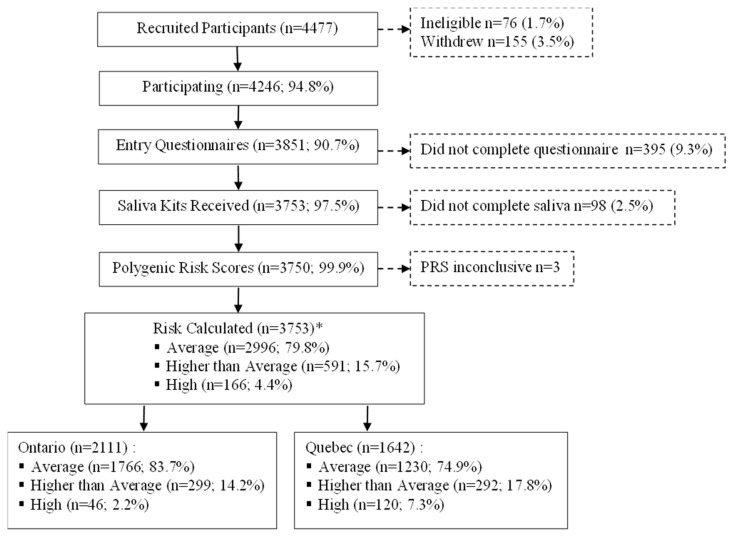
Recruitment and data collection of Quebec and Ontario PERSPECTIVE I&I participants aged 40–69 years. Abbreviations: polygenic risk score (PRS). * Risk estimated without PRS for *n* = 3.

Among the 4246 eligible and consenting participants, 493 (11.6%) did not complete the entry questionnaire or provide a saliva sample and were therefore excluded (Figure 1). Among the remaining 3753 participants, 68 (1.8%) saliva samples were tested and returned inconclusive PRS results. Of the 66 repeat samples received in the case of participants with inconclusive PRS results, 63 yielded a valid PRS, for an overall inconclusive rate of 0.08%. 

Among the 3753 participants, 14.5% were aged 40–49 years, 42.6% were 50–59 and 42.9% were 60–69 (Table 1). Most reported that they were born in Canada (85.5%), not a visible minority (92.8%), were married or had a common law partner (75.1%), had greater than a high school education (87.1%), were employed (61.0%) and had excellent/very good/good overall health (94.8%). 

Compared with participants in Quebec, Ontario participants were more likely to be older (all categories *p* < 0.0001), born outside of Canada (OR = 7.11, 95% CI = 5.44–9.30), a visible minority (OR = 9.33, 95% CI = 6.00–14.52), have a high school diploma or below (OR = 1.29, 95% CI = 1.05–1.59), not employed or retired (*p* < 0.0001) and report their health as fair/poor (OR = 1.71, 95% CI = 1.25–2.32). 

Significant differences were also observed in breast cancer risk factors across the two study sites. Participants from Quebec were significantly more likely to have an age at menopause < 50 (all categories *p* < 0.02), to be current users of oral contraceptives (OR = 2.32, 95% CI = 1.44–3.75), to be former or current users of menopausal hormone therapy (all categories *p* ≤ 0.001), have an age at first live birth of 20–29 years (all categories *p* < 0.0001) and a higher daily alcohol consumption (all categories *p* < 0.05) (Table 2). Significantly more Quebec participants had a first- and/or second-degree family history of cancer (all categories *p* < 0.005) and extremely dense breasts (OR = 1.30, 95% CI = 1.04–1.63).

Overall, 79.8% of participants were estimated to be at average risk, 15.7% at a higher than average risk and 4.4% at high risk (Figure 1). Significantly more Ontario participants were average risk (83.7% vs. 74.9%), whereas in Quebec, higher proportions of participants were identified as higher than average (17.8% vs. 14.2%) and high risk (7.3% vs. 2.2%).

In Ontario, 27.2% of participants chose to complete their entry questionnaire by paper/telephone instead of online (Table 3). These participants were significantly more likely to be aged 60–69 years (OR = 1.32, 95% CI = 1.04–1.67), born outside of Canada (OR = 1.28, 95% CI = 1.01–1.62), a visible minority (OR = 1.51, 95% CI = 1.10–2.07), single, divorced, separated or widowed (OR = 1.47, 95% CI = 1.17–1.84), have a lower educational attainment (college: OR = 2.18, 95% CI = 1.74–2.74; high school or less: OR = 2.32, 95% CI = 1.73–3.12), and fair/poor health (OR = 2.15, 95% CI = 1.46–3.17). 

In total, 1243 (34.4%) participants required verification of some of their breast cancer risk factor information due to missing or unusual values (Table 4). The most frequently verified elements were related to a family history of cancer, including second contralateral breast cancer diagnoses in relatives, obtaining a missing year of birth and/or age at cancer diagnosis for relatives, and clarifying other family information unrelated to cancer (e.g., twins). Participants who required verification of their breast cancer risk factor information were more likely to be from Quebec (OR = 1.90, 95% CI = 1.62–2.24), visible minorities (OR = 1.46, 95% CI = 1.10–1.95) and to have a lower educational attainment (college: OR = 1.26, 95% CI = 1.08–1.47; high school or below: OR = 1.37, 95% CI = 1.09–1.71).

The total per-participant cost of a multifactorial risk assessment was CAD 315, including CAD 265 to generate the polygenic risk score and CAD 50 for the remaining aspects of risk assessment (i.e., the collection of questionnaire-based risk factors, breast density and the estimation of risk using CanRisk).

Compared to individuals included in the CanPath cohort and Canadian Census, a greater proportion of PERSPECTIVE I&I participants were not visible minorities, had a higher educational attainment and were married/had common-law spouses (Table A1, Table A3 and Table A4). Compared to individuals included in the CanPath cohort, a higher percentage of PERSPECTIVE I&I participants were ever users of oral contraceptives, had an age at menopause ≥ 50 years, were never users of menopausal hormone therapy and had a first-degree family history of breast cancer (Table A2).

## 4. Discussion

The PERSPECTIVE I&I project provides a unique opportunity to evaluate risk-stratified breast screening in two Canadian provinces. The recruitment and risk assessment-related outcomes of the cohort study are reported here. Despite extensive disruption to planned recruitment processes due to the COVID-19 pandemic, 4246 eligible people were recruited to participate. Approximately 80% were estimated to be at average risk for breast cancer, while 16% and 4% were estimated to be at a higher than average and high risk, respectively. Risk factors and sociodemographic characteristics differed across the two study sites. More resource-intensive strategies for collecting risk factor information were required for a substantial proportion of participants.

While nearly all participants provided a DNA sample and had their PRS calculated, approximately 10% were withdrawn because they did not complete the entry questionnaire. This suggests that the burden of providing the detailed breast cancer risk factor information that is required for estimating risk may be a barrier to participation for some. A strong alignment between the tools used for collecting risk factor information and those for estimating risk is critical; however, it is important to consider how the data collection burden may be reduced, to maximize participation in risk assessment overall and for specific underserved populations. 

Approximately 16% of our cohort was classified as ‘higher than average’ risk and recommended to have annual screening with mammography. Approximately 4% were high risk and recommended annual screening with mammography and MRI. In the absence of a multifactorial risk assessment, most of these individuals ages 50–69 would only have been eligible for screening once every two years, and those aged 40–49 would not have been invited to screening. These individuals can be considered under-screened according to their actual risk for breast cancer. Some over-screening may also be occurring among people being screened annually, based on the presence of specific risk factors considered in isolation, who would be classified as average risk when considering all the established breast cancer risk factors incorporated by a multifactorial risk assessment.

Important differences were observed across sites. Quebec had a greater proportion of participants aged 40–49, while Ontario’s sample was more diverse with respect to visible minority group membership, employment and health status. The risk distribution also differed, with more Quebec participants estimated at higher than average or high risk. Despite having slightly lower mean polygenic risk scores, Quebec participants more often had family histories of breast cancer and/or extremely dense breasts. These differences may be partially explained by the distributions of these factors in the underlying target populations but are likely driven primarily by the different methods of recruitment employed across the two sites. Approximately 96% of Ontario’s sample was recruited via a population-based invitation approach, while Quebec participants were convenience-sampled. This likely led to a higher number of people aged 40–49 with strong contributory risk factors self-selecting into the study in Quebec. 

One of the major challenges encountered with the collection of breast cancer risk information was that approximately one-third of participants had to be contacted to verify the information they provided, due to missing or unusual values. While the study’s processes were designed anticipating that some degree of verification would be required, the magnitude of this issue was much greater than expected. Contacting individual participants was possible in the research setting but the feasibility of doing this on a larger scale is low, due to the extensive time and resources required. Ensuring that risk factor questionnaires are well-designed, and participants feel comfortable with providing family history information, including for unaffected relatives, is critical for maximizing the quality of the data collected and ultimately the accuracy of the risk prediction. Further studies of this cohort will evaluate the minimum family history data that are required for an accurate risk stratification.

Health literacy is increasingly recognized as a social determinant of health [28], and there is substantial evidence regarding its importance in the contexts of risk communication [29,30,31] and screening behaviour [32]. While the evidence is limited on the influence of health literacy in the pre-communication phases of risk assessment, it is plausible that having lower levels of literacy and health literacy could negatively impact the ability to provide accurate health information. The participants who required risk factor verification were more likely to be visible minorities and less educated. Some of these or related factors have been associated with lower levels of health literacy [33,34]. As risk assessment matures as a population-based approach, participants will likely be responsible for entering their risk factor information into risk assessment tools. A large Canadian survey demonstrated that only 45% of adults aged 18–59 and 12% aged 60 and over have an adequate level of health literacy [34]. Recent reviews of online breast cancer risk assessment tools, and education materials related to breast cancer risk assessment, demonstrated that most are not accessible to people with lower health literacy [35,36]. It is critical that data collection and risk communication tools apply health literacy principles to ensure broad accessibility, and that sufficient resources are dedicated to supporting participants through all the steps of risk-stratified screening.

Participants who chose to complete the entry questionnaire on paper or over the telephone were more likely to be older, not born in Canada, visible minorities, single, widowed or divorced, have a lower educational attainment and poorer health status. In addition to requiring adequate health literacy, providing risk factor information digitally requires adequate digital literacy, as well as access to a device with a stable internet connection. Disparities in access to cancer screening have already been well established. In Canada, recent immigrants, Indigenous, racialized, rural communities, and individuals with lower incomes are more likely to be overdue for screening [37,38,39,40,41]. The broad application of risk assessment for screening will introduce a more complex clinical screening pathway and a reliance upon evolving digital technologies. Concerted efforts will be required to ensure programs meet the unique needs of underserved populations and that cancer disparities are not widened.

Another challenge was related to the collection of breast density information. Our study revealed that breast density is not systematically recorded on mammogram reports in Canada, even within screening programs. For example, it was sometimes recorded only as ≥75% or ˂75% in Ontario and was missing entirely for 125 Quebec participants, requiring a secondary imaging review by a radiologist. Since this time, breast density reporting was standardized in Ontario according to BI-RADS^®^ classifications. The impacts of the variability within and across screening settings in the methods of measuring and reporting breast density on the accuracy of risk assessment will require consideration.

Completing a multifactorial risk assessment with the PRS cost CAD 315 per participant in our cohort. It is likely that the costs of assessment could be further reduced, when applied in the population setting, by benefitting from greater economies of scale; however, it should be noted that there would be significant additional costs associated with achieving the health system readiness required to incorporate risk assessment into existing processes and for the communication of risk. 

This study has many strengths. These include the large sample with comprehensive participant information, including detailed information on sociodemographic factors, and the ability to leverage the infrastructure of organized screening programs, including high-risk services for participants who were estimated to be above average risk. Another substantial strength was the execution of the study at two sites within unique healthcare settings. This created a natural experiment, whereby various methods and tools for recruitment, the collection of breast cancer risk information, risk assessment procedures and risk communication were tested. The ability to test these different methods will generate critical evidence to support planning for implementation on a larger scale. There were some limitations, which may affect the external validity of the study. While a moderate comparability to other Canadian samples was demonstrated, most study participants were white, highly educated and employed. Quebec participants also had to complete their entry questionnaire online. For these reasons, our results are likely affected by healthy user bias. For example, given the stronger preferences expressed for non-digital data collection methods among participants who were visible minorities and had a lower educational attainment, and the under-representation of these demographics in our study population, the 27% who preferred non-digital forms of data collection may be an underestimate. Quebec participants were also required to have a primary health care provider. A 2022 survey found that 22% of Canadians are without a primary care provider, with the figure closer to 30% in Quebec and higher in racialized and lower income communities [42]. It will be important to develop strategies for risk-stratified screening that do not compete for additional primary care resources within an already constrained system.

## 5. Conclusions

It is critical to understand how the various components of risk-stratified screening perform in real-world settings to determine their feasibility and scalability. This study provides many insights regarding the resources required to prepare the health system for facilitating risk assessment. More research is necessary to optimize risk assessment, including understanding the role of health literacy and strategies to increase participation and data accuracy. Future phases of this study will provide evidence on the acceptance, psychological and emotional impacts, effectiveness and costs of risk stratified screening. Further work is required to ensure that the access to risk assessment for risk-stratified screening is equitable and cancer disparities are not widened.

## Figures and Tables

**Table 1:** Sociodemographic and health characteristics of Quebec and Ontario participants, aged 40–69, who completed risk assessment (*n* = 3753), with odds ratios (ORs) and 95% confidence intervals (CIs) comparing Ontario versus Quebec characteristics.

Characteristics	Total N = 3753	Quebec N = 1642	Ontario N = 2111	Odds Ratio Ontario vs. Quebec (95% CI)	*p*-Value
	N (%) *	N (%) *	N (%) *		
Age at study entry (years)					
40–49	544 (14.5)	532 (32.4)	12 (0.6)	0.02 (0.01 to 0.03)	<0.0001
50–59	1600 (42.6)	657 (40.0)	943 (44.7)	1.00 (Referent)	--
60–69	1609 (42.9)	453 (27.6)	1156 (54.8)	1.78 (1.53 to 2.06)	<0.0001
Born in Canada					
Yes	3191 (85.5)	1574 (96.0)	1617 (77.3)	1.00 (Referent)	--
No	540 (14.5)	65 (4.0)	475 (22.7)	7.11 (5.44 to 9.30)	<0.0001
Missing	22	3	19		
Visible minority					
Not a visible minority	3416 (92.8)	1568 (98.6)	1848 (88.4)	1.00 (Referent)	--
Visible minority	264 (7.2)	22 (1.4)	242 (11.6)	9.33 (6.00 to 14.52)	<0.0001
Do not know/prefer not to answer/missing	73	52	21		
Marital status					
Married/common law	2791 (75.1)	1241 (75.8)	1550 (74.6)	1.00 (Referent)	--
Single/widowed/divorced/separated	925 (24.9)	396 (24.2)	529 (25.4)	1.07 (0.92 to 1.24)	0.38
Prefer not to answer/missing	37	5	32		
Highest level of education					
University Bachelor’s degree or above	1904 (51.2)	816 (49.8)	1088 (52.3)	1.00 (Referent)	--
College/Registered Apprenticeship/trades certificate	1336 (35.9)	647 (39.5)	689 (33.1)	0.80 (0.69 to 0.92)	0.002
High school diploma or below	479 (12.9)	176 (10.7)	303 (14.6)	1.29 (1.05 to 1.59)	0.02
Prefer not to answer/missing	34	3	31		
Employment status					
Employed	2277 (61.0)	1125 (68.8)	1152 (54.9)	1.00 (Referent)	
Not employed	256 (6.9)	86 (5.3)	170 (8.1)	1.93 (1.47 to 2.53)	<0.0001
Retired	1202 (32.2)	425 (26.0)	777 (37.0)	1.79 (1.55 to 2.06)	<0.0001
Prefer not to answer/missing	18	6	12		
Overall health					
Excellent/very good/good	3547 (94.8)	1578 (96.2)	1969 (93.7)	1.00 (Referent)	
Fair/poor	194 (5.2)	62 (3.8)	132 (6.3)	1.71 (1.25 to 2.32)	<0.001
Do not know/missing	12	2	10		

* Percentage excludes missing/prefer not to answer/do not know.

**Table 2:** Breast cancer risk factors of Quebec and Ontario participants, aged 40–69, who completed risk assessment (*n* = 3753), with adjusted odds ratios (ORs) and 95% confidence intervals (CIs) comparing Quebec versus Ontario risk factors.

Risk Factors	Total N = 3753	Quebec N = 1642	Ontario N = 2111	Age-Adjusted Odds Ratio (95% CI) ^†^	*p*-Value
	N (%) *	N (%) *	N (%) *		
Height (cm)					
≤152.90	209 (5.6)	79 (4.8)	130 (6.2)	0.78 (0.56 to 1.08)	0.13
152.91–159.64	711 (19.0)	327 (19.9)	384 (18.2)	1.10 (0.90 to 1.34)	0.35
159.65–165.95	1614 (43.1)	747 (45.5)	867(41.1)	1.00 (Referent)	--
165.96–172.69	842 (22.5)	359 (21.9)	483 (22.9)	0.72 (0.59 to 0.88)	0.001
≥172.70	373 (9.9)	130 (7.9)	243 (11.5)	0.46 (0.35 to 0.61)	<0.0001
Missing	4	0	4		
BMI (kg/m^2^)					
<18.5	60 (1.6)	24 (1.5)	36 (1.7)	0.77 (0.41 to 1.46)	0.43
18.5–<25.0	1673 (44.7)	721 (43.9)	952 (45.1)	0.96 (0.80 to 1.16)	0.70
25.0–<30.0	1130 (30.2)	513 (31.2)	617 (29.2)	1.15 (0.94 to 1.40)	0.18
≥30.0	876 (23.5)	384 (23.4)	492 (23.3)	1.00 (Referent)	--
Missing	14	0	14		
Age at menarche (years)					
<11	198 (5.5)	99 (6.3)	99 (4.8)	1.29 (0.91 to 1.83)	0.15
11	479 (13.2)	224 (14.2)	255 (12.4)	1.17 (0.91 to 1.50)	0.22
12	1004 (27.6)	439 (27.9)	565 (27.5)	1.00 (Referent)	--
13	1011 (27.8)	384 (24.4)	627 (30.5)	0.86 (0.70 to 1.05)	0.13
14	557 (15.3)	263 (16.7)	294 (14.3)	1.25 (0.99 to 1.58)	0.07
15	219 (6.0)	101 (6.4)	118 (5.7)	1.27 (0.92 to 1.75)	0.16
>15	164 (4.5)	66 (4.2)	98 (4.8)	0.91 (0.63 to 1.33)	0.63
Missing	121	66	55		
Menopausal status					
Premenopausal	895 (23.9)	643 (39.3)	252 (11.9)	1.09 (0.88 to 1.36)	0.44
Menopausal	2852 (76.1)	994 (60.7)	1858 (88.1)	1.00 (Referent)	--
Missing	6	5	1		
Age at menopause (years)					
<40	185 (7.1)	91 (10.4)	94 (5.4)	2.16 (1.50 to 3.11)	<0.0001
40–44	204 (7.9)	90 (10.4)	114 (6.6)	1.61 (1.13 to 2.32)	0.009
45–49	508 (19.5)	194 (22.2)	314 (18.2)	1.41 (1.07 to 1.86)	0.02
50–54	1239 (47.7)	371 (42.8)	868 (50.2)	1.07 (0.84 to 1.36)	0.61
≥55	464 (17.8)	126 (14.3)	338 (19.6)	1.00 (Referent)	
Missing	252	122	130		
Oral contraceptive use					
Never	377 (10.2)	108 (6.6)	269 (13.1)	0.57 (0.44 to 0.73)	<0.0001
Former	3145 (85.0)	1383 (84.3)	1762 (85.6)	1.00 (Referent)	--
Current	177 (4.8)	149 (9.1)	28 (1.4)	2.33 (1.44 to 3.75)	<0.001
Missing	54	2	52		
Menopausal hormone therapy use					
Never	1889 (66.9)	565 (57.0)	1324 (72.3)	1.00 (Referent)	--
Former (any type)	358 (12.7)	125 (12.6)	233 (12.7)	1.51 (1.18 to 1.92)	0.001
Current (E-type)	272 (9.6)	116 (11.7)	156 (8.5)	1.83 (1.40 to 2.38)	<0.0001
Current (other/unknown type(including combined type))	304 (10.8)	185 (18.7)	119 (6.5)	3.80 (2.95 to 4.91)	<0.0001
Unspecified/missing	29	3	26		
Parity (number of live births)					
Nulliparous	816 (21.7)	306 (18.6)	510 (24.2)	1.00 (Referent)	--
1 birth	565 (15.1)	257 (15.7)	308 (14.6)	1.24 (0.97 to 1.58)	0.08
2 births	1626 (43.3)	749 (45.6)	877 (41.5)	1.22 (1.01 to 1.48)	0.04
>2 births	746 (19.9)	330 (20.1)	416 (19.7)	1.19 (0.95 to 1.49)	0.13
Missing	0	0	0		
Age at first live birth (years)					
<20	100 (3.4)	27 (2.0)	73 (4.6)	0.64 (0.37 to 1.11)	0.11
20–24	549 (18.7)	294 (22.0)	255 (15.9)	2.37 (1.88 to 3.00)	<0.0001
25–29	1178 (40.1)	587 (43.9)	591 (36.9)	1.82 (1.50 to 2.20)	<0.0001
≥30	1110 (37.8)	428 (32.0)	682 (42.6)	1.00 (Referent)	--
Missing	0	0	0		
Alcohol intake per day (grams)					
0	453 (12.7)	141 (8.9)	312 (15.8)	1.00 (Referent)	--
>0–<5	1219 (34.2)	498 (31.3)	721 (36.6)	1.44 (1.11 to 1.87)	0.006
5–<15	1167 (32.8)	611 (38.4)	556 (28.2)	2.33 (1.80 to3.03)	<0.0001
15–<25	340 (9.5)	187 (11.7)	153 (7.8)	2.79 (2.01 to 3.86)	<0.0001
25–<35	229 (6.4)	85 (5.3)	144 (7.3)	1.47 (1.01 to 2.14)	0.04
35–<45	91 (2.6)	36 (2.3)	55 (2.8)	1.73 (1.04 to 2.89)	0.04
≥45	63 (1.8)	35 (2.2)	28 (1.4)	3.23 (1.81 to 5.77)	<0.0001
Missing	191	49	142		
Family history of breast, ovarian, pancreatic and prostate cancer					
First- and second-degree	615 (16.4)	362 (22.0)	253 (12.0)	2.16 (1.74 to 2.69)	<0.0001
First-degree only	701 (18.7)	289 (17.6)	412 (19.5)	1.33 (1.09 to 1.64)	0.006
Second-degree only	1005 (26.8)	502 (30.6)	503 (23.8)	1.56 (1.30 to 1.88)	<0.0001
None	1432 (38.2)	489 (29.8)	943 (44.7)	1.00 (Referent)	--
Family history of any breast cancer					
First- and second-degree	314 (8.4)	213 (13.0)	101 (4.8)	3.06 (2.31 to 4.05)	<0.0001
First-degree only	542 (14.4)	259 (15.8)	283 (13.4)	1.41 (1.14 to 1.75)	0.002
Second-degree only	830 (22.1)	439 (26.7)	391 (18.5)	1.64 (1.36 to 1.97)	<0.0001
None	2067 (55.1)	731 (44.5)	1336 (63.3)	1.00 (Referent)	--
BIRADS^®^ density					
a, b, or c	3232 (87.4)	1333 (83.9)	1899 (90.0)	1.00 (Referent)	--
d	468 (12.6)	256 (16.1)	212 (10.0)	1.30 (1.04 to 1.63)	0.02
Unknown	53	53	0		
Polygenic risk score					
−3.831 to −1.223	374 (10.0)	167 (10.2)	207 (9.8)	1.31 (0.94 to 1.83)	0.11
−1.222 to 0.141	1503 (40.0)	671 (40.9)	832 (39.4)	1.34 (1.02 to 1.74)	0.03
0.142 to 1.477	1498 (39.9)	654 (39.8)	844 (40.0)	1.29 (0.99 to 1.69)	0.06
1.478 to 3.805	375 (10.0)	149 (9.1)	226 (10.7)	1.00 (Referent)	--
Missing	3	1	2		

* Percentage excludes missing/prefer not to answer/do not know. ^†^ Odds ratio adjusted for age, comparing Ontario versus Quebec breast cancer risk factors with ‘Ontario’ as the reference population.

**Table 3:** Sociodemographic and health characteristics of Ontario participants, aged 40–69, who completed risk assessment, by mode of entry questionnaire completion (*n* = 2111), with adjusted odds ratios (ORs) and 95% confidence intervals (CIs) for completing questionnaire on paper/phone versus online.

Characteristics	Total N = 2111	Online N = 1537 (72.8%)	Paper/Phone N = 574 (27.2%)	Adjusted Odds Ratio (95% CI) ^†^	*p*-Value
	N (%) *	N (%) *	N (%) *		
Age at study entry (years)					
40–49	12 (0.6)	7 (0.5)	5 (0.9)	2.79 (0.75 to10.34)	0.13
50–59	943 (44.7)	726 (47.2)	217 (37.8)	1.00 (Referent)	--
60–69	1156 (54.8)	804 (52.3)	352 (61.3)	1.32 (1.04 to 1.67)	0.02
Born in Canada					
Yes	1617 (77.3)	1198 (78.6)	419 (73.9)	1.00 (Referent)	--
No	475 (22.7)	327 (21.4)	148 (26.1)	1.28 (1.01 to 1.62)	0.04
Missing	19	12	7	--	
Visible minority					
Not a visible minority	1848 (88.4)	1368 (89.7)	480 (85.0)	1.00 (Referent)	--
Visible minority	242 (11.6)	157 (10.3)	85 (15.0)	1.51 (1.10 to 2.07)	0.01
Do not know/prefer not to answer/missing	21	12	9		
Marital status					
Married/common law	1550 (74.6)	1167 (76.5)	383 (69.1)	1.00 (Referent)	--
Single/widowed/divorced/separated	529 (25.4)	358 (23.5)	171 (30.9)	1.47 (1.17 to 1.84)	0.001
Prefer not to answer/missing	32	12	20		
Highest level of education					
University Bachelor’s degree or above	1088 (52.3)	881 (57.7)	207 (37.4)	1.00 (Referent)	--
College/Registered Apprenticeship/trades certificate	689 (33.1)	456 (29.9)	233 (42.1)	2.18 (1.74 to 2.74)	<0.0001
High school diploma or below	303 (14.6)	189 (12.4)	114 (20.6)	2.32 (1.73 to 3.12)	<0.0001
Prefer not to answer/missing	31	11	20		
Employment status					
Employed	1152 (54.9)	868 (56.7)	284 (50.0)	1.00 (Referent)	--
Not employed	170 (8.1)	108 (7.1)	62 (10.9)	1.26 (0.86 to 1.84)	0.24
Retired	777 (37.0)	555 (36.3)	222 (39.1)	1.02 (0.80 to 1.30)	0.90
Prefer not to answer/missing	12	6	6		
Overall health					
Excellent/very good/good	1969 (93.7)	1466 (95.6)	503 (88.6)	1.00 (Referent)	--
Fair/poor	132 (6.3)	67 (4.4)	65 (11.4)	2.15 (1.46 to 3.17)	<0.001
Do not know/missing	10	4	6		

* Percentage excludes missing/prefer not to answer/do not know. ^†^ Odds ratio adjusted for age at study entry, visible minority (except born in Canada), marital status, highest level of education, employment status and overall health.

**Table 4:** Sociodemographic characteristics of Quebec and Ontario participants, aged 40–69, who completed risk assessment by entry questionnaire verification status (*n* = 3618), with adjusted odds ratio (ORs) and 95% confidence intervals (CIs) comparing those who required verification and those who did not.

Characteristics	Total N = 3618 *	No Verification N = 2375 (65.6%)	Verification Required N = 1243 (34.4%)	Adjusted Odds Ratio (95% CI) ^‡^	*p*-Value
	N (%) ^†^	N (%) ^†^	N (%) ^†^		
Study Site					
Quebec	1600 (44.2)	935 (39.4)	665 (53.5)	1.90 (1.62 to 2.24)	<0.0001
Ontario	2018 (55.8)	1440 (60.6)	578 (46.5)	1.00 (Referent)	--
Age at study entry (years)					
40–49	531 (14.7)	306 (12.9)	225 (18.1)	1.07 (0.86 to 1.34)	0.54
50–59	1526 (42.2)	1018 (42.9)	508 (40.9)	1.00 (Referent)	--
60–69	1561 (43.1)	1051 (44.3)	510 (41.0)	1.14 (0.95 to 1.36)	0.16
Born in Canada					
Yes	3094 (86.0)	2023 (85.6)	1071 (86.7)	1.00 (Referent)	--
No	503 (14.0)	339 (14.4)	164 (13.3)	1.14 (0.92 to 1.41)	0.24
Missing	21	13	8		
Visible minority					
Not a visible minority	3305 (93.2)	2179 (93.6)	1126 (92.5)	1.00 (Referent)	--
Visible minority	241 (6.8)	150 (6.4)	91 (7.5)	1.46 (1.10 to 1.95)	0.009
Do not know/prefer not to answer/missing	72	46	26		
Marital status					
Married/common law	2691 (75.0)	1774 (75.4)	917 (74.4)	1.00 (Referent)	--
Single/widowed/divorced/separated	896 (25.0)	580 (24.6)	316 (25.6)	1.03 (0.88 to 1.22)	0.70
Prefer not to answer/missing	31	21	10		
Highest level of education					
University Bachelor’s degree or above	1831 (51.1)	1254 (53.1)	577 (47.1)	1.00 (Referent)	--
College/Registered Apprenticeship/trades certificate	1292 (36.0)	816 (34.6)	476 (38.9)	1.26 (1.08 to 1.47)	0.0030.003
High school diploma or below	462 (12.9)	290 (12.3)	172 (14.0)	1.37 (1.09 to 1.71)	0.006
Prefer not to answer/missing	33	15	18		
Employment status					
Employed	2186 (60.7)	1418 (60.0)	768 (62.2)	1.00 (Referent)	--
Not employed	246 (6.8)	143 (6.0)	103 (8.3)	1.29 (0.97 to 1.71)	0.08
Retired	1168 (32.4)	804 (34.0)	364 (29.5)	0.84 (0.70 to 1.01)	0.07
Prefer not to answer/missing	18	10	8		
Overall health					
Excellent/very good/good	3424 (94.9)	2263 (95.4)	1161 (93.9)	1.00 (Referent)	--
Fair/poor	183 (5.1)	108 (4.6)	75 (6.1)	1.30 (0.95 to 1.78)	0.11
Do not know/missing	11	4	7		

* Excludes pilot study participants (*n* = 135); ^†^ Percentage excludes missing/prefer not to answer/do not know; ^‡^ Odds ratio adjusted for study site, age at study entry, visible minority (except born in Canada), marital status, highest level of education, employment status and overall health.

## Data Availability

Parts of the material underlying this article are based on data and information provided by Ontario Health (Cancer Care Ontario). Ontario Health is prohibited from making the data used in this research publicly accessible if it includes potentially identifiable personal health information and/or personal information as defined in Ontario law, specifically the Personal Health Information Protection Act (PHIPA) and the Freedom of Information and Protection of Privacy Act (FIPPA). Upon request, data de-identified to a level suitable for public release may be provided.

## Appendix A

### Appendix A.1. Supplementary Methods

#### Appendix A.1.1. Alcohol Intake

Alcohol intake was classified as 0, >0 to <5, 5 to <15, 15 to <25, 25 to <35, 35 to <45, or ≥45 g per day, based on Canadian standard drink volumes. Calculations for grams per day of alcohol were based on the type of drink, strength (given by alcohol by volume [ABV] and volume), as follows:

**Type of Drink**	**Strength (ABV %)**	**Volume (mL)**
Bottle of beer, cider or cooler	5	341
Pint of cider or beer	4	586
Glass of wine	11	142
Shot of liquor	40	43

The following shows an example for one standard glass (142 mL) of 11% ABV wine daily.

(a) Calculate the units: strength (ABV) × volume (mL)/1000 = units
  - 11 × 142/1000 = 1.562 units

(b) Calculate the grams of alcohol: assume that 1 unit = 8 g of alcohol
  - 1.562 × 8 = 12.5 g

(c) Calculate grams per day based on the reported number of drinks and frequency (daily, weekly, monthly)
  a. Daily = 12.5 g
  b. Weekly (divide by 7) = 1.8 g/day
  c. Monthly (divide by 30) = 0.4 g/day

#### Appendix A.1.2. Calculation of Standardized Difference

For dichotomous variables, the standardized difference is defined as:

$$d=\frac{\left(\hat{p}treatment-\hat{p}control\right)}{\sqrt{\;\frac{\hat{p}treatment\;\left(1-\hat{p}treatment\right)+\hat{p}control\;\left(1-\hat{p}control\right)}{2}\;\;\;\;\;\;}\;\;\;}$$

where $\hat{p}treatment$ and $\hat{p}control$ denote the prevalence or mean of the dichotomous variable in treated and untreated subjects, respectively. In our study, the treatment group was the PERSPECTIVE I&I cohort and the control group, the population-based data (CanPath and Census).

**Table A1:** Sociodemographic characteristics of PERSEPCTIVE I&I participants, aged 40–69, compared to participants in CanPath (Ontario and Quebec; Canada) aged 40–69, who ever had a mammogram.

Characteristics	PERSPECTIVE I&I Participants N = 3753	CanPath Ontario– Quebec Participants * N = 72,314	Standardized Difference (%)	CanPath Canada Participants * N = 114,599	Standardized Difference (%)
	N (%) ^†^	N (%) ^†^		N (%) ^†^	
Age at study entry (years)					
40–49	544 (14.5)	26,949 (37.3)	−53.9	38,773 (33.8)	−46.3
50–59	1600 (42.6)	28,657 (39.6)	6.1	45,673 (39.9)	5.5
60–69	1609 (42.9)	16,708 (23.1)	43.1	30,153 (26.3)	35.4
Born in Canada					
Yes	3191 (85.5)	55,040 (81.8)	10.0	88,326 (82.6)	7.9
No	540 (14.5)	12,266 (18.2)	−10.0	18,555 (17.4)	−7.9
Missing	22	5008		7718	
Visible minority					
Not a visible minority ^‡^	3416 (92.8)	56,393 (88.9)	13.6	89,798 (90.8)	7.3
Visible minority	264 (7.2)	7057 (11.1)	−13.6	9058 (9.2)	−7.3
Do not know/missing	73	8864		15,743	
Race/ethnicity					
Black	35 (1.0)	817 (1.3)	−2.8	912 (0.9)	1.0
East Asian	92 (2.5)	1730 (2.7)	−1.7	2451 (2.5)	0.0
Indigenous	43 (1.2)	1645 (2.6)	−10.3	2801 (2.8)	−11.5
Latin American/Hispanic	41 (1.1)	414 (0.7)	4.2	544 (0.6)	5.5
Arab	5 (0.1)	326 (0.5)	−7.3	360 (0.4)	−6.0
West Asian	11 (0.3)	93 (0.1)	4.5	157 (0.2)	2.0
South Asian	24 (0.7)	801 (1.3)	−6.0	979 (1.0)	−3.3
Southeast Asian	30 (0.8)	440 (0.7)	1.2	610 (0.6)	2.4
White	3361 (91.3)	54,577 (86.0)	16.8	86,718 (87.7)	11.8
Other/Mixed ^§^	38 (1.0)	2607 (4.1)	−19.8	3324 (3.4)	−16.4
Do not know/missing	73	8864		15,743	
Marital status					
Married/common law	2791 (75.1)	49,901 (69.2)	13.2	79,149 (69.8)	11.9
Single/widowed/divorced/separated	925 (24.9)	22,237 (30.8)	−13.2	34,194 (30.2)	−11.9
Missing	37	176		1256	
Highest level of education					
University Bachelor’s degree or above	1904 (51.2)	26,409 (36.9)	29.1	41,664 (36.7)	29.5
College/Registered Apprenticeship/trades certificate	1336 (35.9)	28,715 (40.1)	−8.7	45,681 (40.3)	−9.1
High school diploma or below	479 (12.9)	16,424 (23.0)	−26.6	26,034 (23.0)	−26.6
Prefer not to answer/missing	34	766		1220	
Employment status **					
Employed	2277 (61.0)	44,248 (63.2)	−4.5	70,001 (63.1)	−4.3
Not employed/retired	1458 (39.0)	25,808 (36.8)	4.5	40,979 (36.9)	4.3
Prefer not to answer/missing	18	2258		3619	

* Includes all people who identified as female who are breast cancer-free aged 40–69 with history of mammogram. ^†^ Percentage includes missing/prefer not to answer/do not know. ^‡^ Includes those who are Indigenous, Indigenous and White, White, White and Arab, White and Latin American, White and West Asian. ^§^ Mixed race/ethnicity includes those who reported more than one ethnicity group. ** For CanPath data, a response of working full-time or part-time was included in “employed”; unable to work was categorized as “not employed”, as there were no details whether this was temporary or permanent. There are no options for ‘currently on maternity or parental leave’ in the CanPath dataset.

**Table A2:** Breast cancer risk factors of PERSPECTIVE I&I participants, aged 40–69, compared to participants in CanPath (Ontario and Quebec; Canada) aged 40–69, who ever had a mammogram.

Risk Factors	PERSPECTIVE I&I Participants N = 3753	CanPath Ontario–Quebec Participants * N = 72,314	Standardized Difference (%)	CanPath Canada Participants * N = 114,599	Standardized Difference (%)
	N (%) †	N (%) †		N (%) †	
Height (cm)					
≤152.90	208 (5.6)	2907 (5.9)	−1.3	4073 (5.8)	−0.9
152.91–159.64	711 (19.0)	9205 (18.6)	1.0	13,757 (19.6)	−1.5
159.65–165.95	1614 (43.1)	20,174 (40.9)	4.5	28,339 (40.4)	5.5
165.96–172.69	842 (22.5)	11,112 (22.5)	0.0	16,047 (22.9)	−1.0
≥172.70	372 (9.9)	5986 (12.1)	−7.0	7851 (11.2)	−4.2
Missing ‡	6	22,930		44,532	
BMI (kg/m^2^) §					
<18.5	60 (1.6)	649 (1.5)	0.8	899 (1.4)	1.7
18.5–<25	1673 (44.7)	18,530 (42.6)	4.2	27,170 (42.7)	4.0
25–<30	1128 (30.2)	13,403 (30.8)	−1.3	19,758 (31.1)	−2.0
≥30	878 (23.5)	10,948 (25.2)	−4.0	15,756 (24.8)	−3.0
Missing	14	28,784		51,016	
Age at menarche (years)					
<11	198 (5.5)	4282 (6.4)	−3.8	6241 (5.8)	−1.3
11	479 (13.2)	9223 (13.8)	−1.8	14,365 (13.5)	−0.9
12	1004 (27.6)	17,718 (26.6)	2.3	28,667 (26.8)	1.8
13	1011 (27.8)	18,105 (27.2)	1.3	29,803 (27.9)	−0.2
14	557 (15.3)	9872 (14.8)	1.4	15,771 (14.8)	1.4
15	219 (6.0)	4150 (6.2)	−0.8	6872 (6.4)	−1.7
>15	164 (4.5)	3261 (4.9)	−1.9	5050 (4.7)	−1.0
Missing **	121	5703		7830	
Age at menopause (years) ††					
<40	185 (7.1)	4407 (12.3)	−17.6	7597 (12.6)	−18.5
40–44	204 (7.9)	4267 (11.9)	−13.4	6977 (11.6)	−12.5
45–49	508 (19.6)	8778 (24.5)	−11.8	14,354 (23.8)	−10.2
50–54	1239 (47.8)	14,248 (39.8)	16.2	24,153 (40.1)	15.6
≥55	456 (17.6)	4091 (11.4)	17.7	7104 (11.8)	16.4
Missing ‡‡	260	2737		4511	
Oral contraceptive use					
Never	377 (10.2)	9758 (14.0)	−11.7	13,869 (12.5)	−7.3
Ever §§	3322(89.8)	59,860 (86.0)	11.7	97,392 (87.5)	7.3
Missing	54	2696		3338	
Menopausal hormone therapy use ††					
Never	1889 (66.9)	24,336 (63.6)	6.9	38,980 (60.7)	12.9
Ever	934 (33.1)	13,913 (36.4)	−6.9	25,274 (39.3)	−12.9
Missing	29	279		442	
Parity (number of live births)					
Nulliparous ***	816 (21.7)	13,475 (19.8)	4.7	21,187 (19.5)	5.4
1 birth	566 (15.1)	11,102 (16.3)	−3.3	16,634 (15.3)	−0.6
2 births	1625 (43.3)	27,840 (40.9)	4.9	44,997 (41.3)	4.1
>2 births	746 (19.9)	15,581 (22.9)	−7.3	26,044 (23.9)	−9.7
Missing	0	4316		5737	
Alcohol intake per day (grams)					
0–<5	1672 (46.9)	38,620 (58.4)	−23.2	61,935 (59.6)	−25.7
5–<15	1167 (32.8)	15,381 (23.3)	21.3	24,067 (23.2)	21.5
15–<25	340 (9.5)	6495 (9.8)	−1.0	9519 (9.2)	1.0
25–<35	229 (6.4)	2704 (4.1)	10.3	4052 (3.9)	11.3
35–<45	91 (2.6)	1279 (1.9)	4.7	1924 (1.9)	4.7
≥45	63 (1.8)	1651 (2.5)	−4.8	2425 (2.3)	−3.5
Missing †††	191	6184		10,677	
Breast and ovarian cancer among first-degree relatives ‡‡‡					
Breast cancer only	883 (23.5)	6921(12.2)	29.8	12,042 (13.0)	27.4
Ovarian cancer only	109 (2.9)	1077 (1.9)	6.5	1901 (2.0)	5.8
Both breast and ovarian cancer	28 (0.7)	261 (0.5)	2.6	420 (0.5)	2.6
None	2733 (72.8)	48,440 (85.4)	−31.4	78,600 (84.5)	−28.9
Missing	0	15,615		21,636	

* Includes all people who identified as female who are breast cancer-free aged 40–69 with a history of mammogram. ^†^ Percentage excludes missing/prefer not to answer/do not know. ^‡^ Height < 121.92 cm, i.e., 4 feet, and >213.36 cm, i.e., 7 feet cm, were coded as missing. ^§^ BMI <12.6 kg/m^2^ and >160 kg/m^2^ were coded as missing. ** Age at menarche < 8 years and >24 years were coded as missing. ^††^ Excludes pre-menopause and menopause status missing (PERSPECTIVE *n* = 901; CanPath *n* = 49,903). ^‡‡^ Age at menopause <20 years and >60 years were coded as missing. ^§§^ Former user includes OC ever users (PERSPECTIVE *n* = 15). *** Includes pregnant but no live births and never pregnant (PERSPECTIVE pregnant/no live births *n* = 85; PERSPECTIVE never pregnant *n* = 732; CanPath pregnant/no live births *n* = 4398; CanPath never pregnant *n* = 16,789). ^†††^ Includes ever users whose daily alcohol intake was not calculated (NA) (PERSPECTIVE *n* = 180). ^‡‡‡^ Includes any breast (unilateral and bilateral) and ovarian cancer among all first-degree relatives (male or female) for Ontario and Canada (excluding Quebec).

**Table A3:** Sociodemographic characteristics of PERSPECTIVE I&I participants, aged 40–69, compared to participants in Census (Canada).

Characteristics	PERSPECTIVE Total	Census * Canada	Standardized Difference (%)
	N (%) ^†^	N (%) ^†^	
Age at study entry (years)			
40–49	544 (14.5)	2,398,515 (32.3)	−43.0
50–59	1600 (42.6)	2,554,035 (34.4)	16.9
60–69	1609 (42.9)	2,463,910 (33.2)	20.1
Born in Canada			
Yes	3191 (85.5)	13,580,610 (73.8)	29.4
No	540 (14.5)	4,810,705 (26.2)	−29.4
Missing	22		
Visible minority			
Not a visible minority ^‡^	3416 (92.8)	13,464,900 (73.2)	54.1
Chinese	78 (2.1)	907,035 (4.9)	−15.3
Black	40 (1.1)	793,765 (4.3)	−19.8
Filipino	22 (0.6)	529,600 (2.9)	−17.6
Latin American	41 (1.1)	299,200 (1.6)	−4.3
South Asian	32 (0.9)	1,247,275 (6.8)	−31.0
Multiple visible minorities ^§^	11 (0.3)	171,010 (0.9)	−7.8
Other visible minorities **	40 (1.1)	978,525 (5.3)	−24.0
Do not know/prefer not to answer/missing	73		
Marital status ^††^			
Married/common law	2791 (75.1)	8,812,595 (55.6)	41.9
Single/widowed/divorced/separated	925 (24.9)	7,026,860 (44.4)	−41.9
Missing	37		
Highest level of education ^‡‡^			
University Bachelor’s degree or above	1904 (51.2)	3,594,215 (36.1)	30.8
College/Registered Apprenticeship/trades certificate	1336 (35.9)	3,364,885 (33.8)	4.4
High school diploma or below	479 (12.9)	2,988,825 (30.0)	−42.6
Prefer not to answer/missing	34		
Employment status ^§§^			
Employed	2277 (61.0)	8,265,025 (53.4)	15.4
Not employed/retired	1458 (39.0)	7,209,650 (46.6)	−15.4
Prefer not to answer/missing	18		

* Includes all people who identified as female or non-binary from the 2021 Census (information on mammogram history is not available in the Census). ^†^ Percentage excludes missing/prefer not to answer/do not know. ^‡^ Includes those who identified as Indigenous (Total *n* = 43; Ontario *n* = 25; Quebec *n* = 18). ^§^ Includes those with two or more visible minority memberships (e.g., Black and Chinese) and those with White and two or more visible minority memberships (e.g., White, Arab and Chinese) ** Includes those with one visible minority membership only not captured in previous categories (Korean, Japanese, Arab, West Asian and Southeast Asian). ^††^ For total population aged 15 years and older. ^‡‡^ For the population aged 25 to 64 years in private households; 25% sample data. ^§§^ For the population aged 15 years and over by work activity during the reference year; 25% sample data.

**Table A4:** Sociodemographic characteristics of PERSPECTIVE I&I participants, aged 40–69, compared to participants in Census (Ontario and Quebec).

Characteristics	PERSPECTIVE Ontario	Census * Ontario	Standardized Difference (%)	PERSPECTIVE Quebec	Census * Quebec	Standardized Difference (%)
	N (%) †	N (%) †		N (%) †	N (%) †	
Age at study entry (years)						
40–49	12 (0.6)	926,300 (32.3)	−94.6	532 (32.4)	543,065 (31.8)	1.3
50–59	943 (44.7)	1,018,310 (35.5)	18.9	657 (40.0)	568,790 (33.3)	13.9
60–69	1156 (54.8)	925,515 (32.2)	46.8	453 (27.6)	595,850 (34.9)	−15.8
Born in Canada						
Yes	1617 (77.3)	4,745,495 (66.4)	24.4	1574 (96.0)	3,467,705 (82.9)	43.7
No	475 (22.7)	2,398,690 (33.6)	−24.4	65 (4.0)	713,915 (17.1)	−43.7
Missing	19			3		
Visible minority						
Not a visible minority ‡	1848 (88.4)	4,676,955 (65.5)	56.5	1568 (98.6)	3,502,955 (83.8)	54.1
Chinese	78 (3.7)	431,935 (6.0)	−10.7	≤5 (0.0)	63,865 (1.5)	−17.5
Black	34 (1.6)	402,940 (5.6)	−21.6	6 (0.4)	216,070 (5.2)	−5.7
Filipino	22 (1.1)	206,070 (2.9)	−12.9	≤5 (0.0)	25,580 (0.6)	−11.0
Latin American	27 (1.3)	129,830 (1.8)	−4.1	14 (0.9)	87,880 (2.1)	30.5
South Asian	32 (1.5)	736,020 (10.3)	−38.0	≤5 (0.0)	60,850 (1.5)	−17.5
Multiple visible minorities §	11 (0.5)	93,750 (1.3)	−8.5	≤5 (0.0)	17,875 (0.4)	−9.0
Other visible minorities **	38 (1.8)	466,685 (6.5)	−23.7	2 (0.1)	206,545 (4.9)	−23.2
Do not know/prefer not to answer/missing	21			52		
Marital status ††						
Married/common law	1550 (74.6)	3,385,240 (55.0)	41.9	1241 (75.8)	1,981,145 (54.7)	45.4
Single/widowed/divorced/separated	529 (25.4)	2,771,690 (45.0)	−41.9	396 (24.2)	1,640,660 (45.3)	−45.4
Missing	32			5		
Highest level of education ‡‡						
University Bachelor’s degree or above	1088 (52.3)	1,900,925 (31.4)	43.4	816 (49.8)	733,395 (33.0)	34.6
College/Registered Apprenticeship/trades certificate	689 (33.1)	1,657,230 (27.4)	12.4	647 (39.5)	906,335 (40.7)	−2.5
High school diploma or below	303 (14.6)	2,491,315 (41.2)	−62.1	176 (10.7)	586,045 (26.3)	−41.0
Prefer not to answer/missing	31			3		
Employment status §§						
Employed	1152 (54.9)	3,075,940 (50.8)	8.2	1125 (68.8)	1,974,095 (56.3)	26.0
Not employed/retired	947 (45.1)	2,973,520 (49.2)	−8.2	511 (31.2)	1,530,115 (43.7)	−26.0
Prefer not to answer/missing	12			6		

* Includes all people who identified as female or non-binary from the 2021 Census (information on mammogram history is not available in the Census). ^†^ Percentage excludes missing/prefer not to answer/do not know. ^‡^ Includes those who identified as Indigenous (Total *n* = 43; Ontario *n* = 25; Quebec *n* = 18). ^§^ Includes those with two or more visible minority memberships (e.g., Black and Chinese) and those with White and two or more visible minority memberships (e.g., White, Arab and Chinese) ** Includes those with one visible minority membership only not captured in previous categories (Korean, Japanese, Arab, West Asian and Southeast Asian). ^††^ For total population aged 15 years and older. ^‡‡^ For the population aged 25 to 64 years in private households; 25% sample data. ^§§^ For the population aged 15 years and over by work activity during the reference year; 25% sample data.
